# Between Patient Pressure and Professional Responsibility: Antibiotic Prescribing Practices in Primary Care

**DOI:** 10.3390/healthcare14111506

**Published:** 2026-05-29

**Authors:** Nóra Horváth, Csongor István Szepesi, Viktor Rekenyi, Anna Nánási, Eszter Kovács, László Róbert Kolozsvári

**Affiliations:** 1 Doctoral School of Health Sciences, University of Debrecen, 4032 Debrecen, Hungary; 2 Department of Family and Occupational Medicine, Faculty of Medicine, University of Debrecen, 4032 Debrecen, Hungary

**Keywords:** antimicrobial resistance, general practitioners, primary healthcare, antibiotic prescribing, patient expectations

## Abstract

**Highlights:**

**What are the main findings?**

- In total, 80.6% of GPs report patients expecting antibiotics; 87.2% resist pressure to prescribe.
- In total, 56.1% of GPs prescribed complete patient-initiated antibiotic courses at least once within the last 6 months.
- Only 17.8% of GPs regularly monitor antibiotic prescribing indicators, limiting stewardship efforts.
- Physician specialty qualifications correlate positively with antibiotic knowledge but inversely with patient education.

**What are the implications of the main findings?**

- Antimicrobial stewardship efforts must extend beyond prescriber-focused interventions and address patient-driven behaviors in primary care.
- System-level strategies are needed to reduce inappropriate antibiotic initiation outside the clinical encounter and to strengthen public health education.

**Abstract:**

**Background/Objectives:** Antimicrobial resistance (AMR) represents one of the most pressing global public health challenges, with inappropriate antibiotic prescribing being a major contributor. In Hungary, general practitioners (GPs) account for over 70% of all antibiotic prescriptions, yet limited research has examined the complex relationship between patient expectations and physician prescribing behavior. This study explores general practitioners’ antibiotic prescribing practices and their perceptions of patient expectations. **Methods:** A cross-sectional study was conducted among 181 GPs in Hungary from March 2024 to April 2025. The sample is representative of the northeastern region of Hungary. Participants completed anonymous paper-based questionnaires assessing their self-reported professional knowledge, perceived patient expectations, prescribing behavior, and antibiotic stewardship practices. **Results:** Most respondents recognized antimicrobial resistance as a significant public health issue (81.7%, n = 147); however, only 52.2% (n = 94) felt capable of effectively taking action against it. While 80.6% (n = 145) reported that patients expect antibiotic prescriptions and 71.1% (n = 128) experienced conflicts over prescribing refusals at least once within the previous six months, 87.2% (n = 157) stated they do not yield to patient pressure. Concerning patterns emerged: 56.1% (n = 101) reported completing patient-initiated antibiotic courses, 36.1% (n = 65) admitted to “just-in-case” prescribing at least once within the past six months, and 38.9% (n = 70) encountered self-medication despite regulations restricting antibiotics to prescription-only use. Only 17.8% (n = 32) regularly monitored their antibiotic prescribing indicators. Physicians with multiple specialty qualifications reported less frequent patient education and more conflicts (*p* = 0.010). **Conclusions:** General practitioners demonstrate resilience despite substantial patient pressure; however, self-medication and defensive prescribing practices reveal important gaps in antimicrobial stewardship. Targeted, multifaceted interventions addressing both prescriber behavior and systemic vulnerabilities are needed to strengthen stewardship efforts.

## 1. Introduction

### 1.1. Understanding Antimicrobial Resistance

Antimicrobial resistance (AMR) arises naturally as pathogens undergo genetic changes over time. However, human activities, especially the inappropriate and excessive use of antimicrobials to treat, prevent, or manage infections in humans, animals, and plants, significantly accelerate the development and dissemination of resistance [1].

This phenomenon, identified across all pathogen types, is particularly concerning regarding bacterial antibiotic resistance, as these organisms can rapidly develop immunity to numerous new antibiotics intended to combat bacterial infections [2].

### 1.2. The Evolution and Scope of AMR

While drug-resistant infections were once mostly confined to hospitals and healthcare facilities, they have increasingly appeared in the broader community over the past decade. As resistance continues to rise, the substantial progress made in infectious disease control over the last century is at risk of being undone [3].

AMR represents one of the most pressing global public health challenges of the 21st century, with inappropriate antibiotic prescribing serving as a primary driver of this phenomenon [4,5]. By 2050, if no preventive interventions are implemented, antimicrobial resistance is predicted to become the leading cause of death worldwide [3].

In 2019, an estimated 4.95 million deaths were associated with drug-resistant infections, with 1.27 million deaths directly attributable to AMR. Hungary is similarly affected, with approximately 1600 deaths in 2019 directly linked to antimicrobial-resistant infections, highlighting the substantial national burden of this issue [6].

### 1.3. Being a General Practitioner in Hungary: System Structure and Challenges

The system is based on “territorial care obligation,” where most GP practices are assigned defined geographic areas and must provide care to residents within them. A smaller number of practices operate without such obligations, allowing for more flexibility in patient selection. Despite territorial assignments, Hungarian law ensures free choice of physician, meaning patients may register with any GP who agrees to accept them, even outside their designated area.

Financing includes performance-based incentives to encourage rational antibiotic use through the indicator system. The National Health Insurance Fund (NEAK) monitors prescribing patterns at the territorial level, and GPs exceeding set thresholds in a given month may face funding reductions. This approach supports national efforts to combat antimicrobial resistance through financial incentives [7].

Hungary also operates an extensive on-call system for after-hours care. However, some patients misuse these services to bypass their regular GP, particularly when seeking antibiotics that might otherwise be refused [8]. This highlights the challenge of balancing accessible care with appropriate prescribing and antimicrobial stewardship in primary healthcare [9,10].

### 1.4. Primary Care as the Main Setting for Antibiotic Prescribing

Globally, the majority of antibiotics prescribed for humans occur in outpatient primary care settings. These settings account for approximately 90% of all antibiotic prescriptions in the European Union and 80–90% in the United States [11,12]. In Hungary specifically, antibiotic prescriptions by general practitioners account for more than 70% of total antibiotic prescriptions [4].

#### 1.4.1. The Hardships of Prescribing Decisions

The complexity of antibiotic prescribing decisions in primary care extends beyond simple clinical considerations, encompassing a multifaceted interplay of physician-related factors, patient expectations, diagnostic uncertainty, and healthcare system constraints [13,14].

Evidence suggests that higher antibiotic use is associated with factors such as insufficient prescriber knowledge, resistance or indifference to change, complacency stemming from patient satisfaction, low household income, and prescribers practicing in rural areas [15].

##### Diagnostic Uncertainty

While viruses are responsible for about 90% of upper respiratory infections, between 50% and 70% of patients seeking medical attention for these illnesses are prescribed antibiotics. Prescribing antibiotics in cases where they offer no proven benefit is far from harmless; in fact, it significantly contributes to the emergence of antibiotic resistance [16,17].

##### Telemedicine

The COVID-19 pandemic also led to a significant increase in the use of telemedicine, prompting concerns regarding its impact on antibiotic prescribing practices. Emerging evidence suggests that remote consultations are associated with higher rates of antibiotic prescriptions compared to in-person visits, particularly for respiratory and urinary tract infections, and especially among younger adults who are more likely to use telehealth services [18].

##### Patient Expectations

A recent systematic review confirms that patient requests are a powerful determinant of physicians’ prescribing behavior [19]. A 2025 study analyzing nearly 5000 primary care consultations across 18 European countries, including contributions from five Hungarian GPs, found that when physicians perceived a patient request for antibiotics, they were 4.4 times more likely to prescribe them compared with consultations without such perceived demand. This perceived patient expectation emerged as one of the strongest determinants of antibiotic prescribing, second only to the GP’s assessment of illness severity. Notably, one of the highest prescribing rates were observed in Hungary, where antibiotics were prescribed in 52% of primary care consultations for respiratory tract infections [20].

Data from 2013 demonstrate comparatively high levels of patient expectation and antibiotic prescribing in Hungarian primary care consultations for acute cough and lower respiratory tract infections (LRTIs). More than half of patients reported expecting (50.3%) or hoping for (54.4%) antibiotics, while 31.9% explicitly requested them. Despite these expectations, overall patient satisfaction remained high, with 96.3% reporting being satisfied or very satisfied with the consultation. Clinicians perceived patient demand for antibiotics in 41.9% of encounters, representing one of the highest proportions among participating countries. Antibiotics were prescribed in 74.7% of cases, one of the highest rates observed across the European primary care networks studied [21]. These findings are particularly concerning given that patient demand has been identified as one of the strongest determinants of antibiotic prescribing in such clinical contexts. Nevertheless, patient expectations and their impact on prescribing behavior remain insufficiently studied in Hungary.

## 2. Objective

The objective of this study was to investigate primary care physicians’ perceptions of patient expectations and pressure regarding antibiotics, and to evaluate their self-reported antibiotic prescribing behaviors and antimicrobial stewardship practices in Hungarian primary care.

## 3. Methods

### 3.1. Study Design and Setting

A cross-sectional study was conducted among physicians in Hungary between 24 March 2024, and 20 April 2025. This study was designed and reported in accordance with the Strengthening the Reporting of Observational Studies in Epidemiology (STROBE) guidelines [22]. Data collection was centered at the Department of Family and Occupational Medicine at the University of Debrecen, Faculty of Medicine, which serves as the designated regional hub for organizing mandatory Continuing Medical Education courses for the northeastern region of Hungary. These courses are a statutory requirement for all GPs every five years to maintain their medical license, and for GP trainees as a prerequisite for their specialist examinations. This mandatory nature ensured that the sampling frame included a broad cross-section of the professional population, rather than only those physicians who are highly motivated or proactive in voluntary professional development. The study was voluntary and anonymous, and data collection was conducted through the distribution of paper-based questionnaires.

#### 3.1.1. Participants and Eligibility Criteria Exclusion Criteria

Physicians were eligible for inclusion in the study if they were currently holding a GP qualification or were enrolled in a GP specialty training program and were attending the mandatory training. Conversely, individuals were excluded from the final analysis if they failed to provide a signed informed consent form, returned extensively incomplete questionnaires, or were medical professionals from specialties other than general practice.

#### 3.1.2. Representativeness and Sampling Frame

While the courses were open to physicians nationwide, the University of Debrecen primarily serves three counties comprising the northeastern region of Hungary. Within the final sample, 114 participants were active GPs practicing within this specific region. According to the registry data during the research period, there were a total of 776 active GPs in these three northeastern counties [23]. Consequently, this regional sub-sample represents a sampling fraction of 14.69%, demonstrating great population coverage for this geographic area. At a 95% confidence level, this sample size yields a margin of error of ±8.48%. Due to the mandatory nature of the attendance for licensure, the sample is considered representative of the regional GP workforce, effectively mitigating the selection bias typically associated with convenience sampling in voluntary educational settings.

### 3.2. Measures

The questionnaire consisted of four main sections targeting different aspects of antibiotic prescription habits and patient expectations.

The first section gathered demographic data, including age, gender, place of residence and work, year of graduation, medical specialties held by the respondent, and whether they currently manage a general practice, along with the number of registered patients.

The second section assessed professional characteristics, which included physicians’ self-reported knowledge and attitudes toward antibiotics and antimicrobial resistance using Likert-scale statements (1–5), such as perceived adequacy of their professional knowledge of antibiotic therapy, awareness of resistance mechanisms, and perceived responsibility in combating resistance.

The third section focused on patient-related factors, including perceived patient expectations and experiences of pressure to prescribe antibiotics. Physicians were also asked about their own prescribing habits, including the use of antibiotics during telephone consultations and adherence to physical examinations before prescribing.

The last section of the questionnaire further explored the frequency of antibiotic-related events in clinical practice, such as patient conflicts, precautionary prescriptions before holidays or weekends, patient loss due to disagreements over antibiotic use, and physicians complying with completing self-initiated antibiotic regimens, using a scale from “never” to “daily.”

The internal consistency of the questionnaire was acceptable, with an overall Cronbach’s alpha of 0.72. Currently, the instrument is not yet formally validated. It was newly developed specifically for this research to accurately reflect the unique socio-cultural context of the Hungarian primary care system and the specific contemporary challenges GPs face regarding antibiotic prescribing, nuances that existing standardized international tools may not sufficiently capture. Consequently, this study serves as the critical first step toward the psychometric evaluation and future formal validation of the questionnaire.

### 3.3. Data Categorization Approach

Given the nature of the collected data, several levels of categorization were implemented for our analysis.

In the second part of the questionnaire, physicians’ self-reported knowledge and attitudes toward antibiotics and antimicrobial resistance were measured using a Likert-scale format with responses ranging from 1 to 5. For analysis, these responses were categorized into three groups: those scoring 5 points, those scoring 4 points, and those scoring “low,” meaning scores between 1 and 3. This three-level categorization allowed a nuanced distinction between very high, moderately high, and lower confidence participants.

In the third part of the questionnaire, where physicians rated statements on a four-part Likert scale (“Not at all characteristic,” “Slightly characteristic,” “Moderately characteristic,” and “Very characteristic”) responses were dichotomized into “Characteristic” (combining “Moderately” and “Very characteristic”) and “Not Characteristic” (combining “Not at all” and “Slightly characteristic”). This binary grouping facilitated clearer interpretation of whether the behavior or attitude was substantively present.

Finally, for the frequency of antibiotic-related events, measured on a seven-point Likert scale ranging from “Never” to “Daily,” the responses were collapsed into two groups: events occurring less frequently than every 6 months (including “Never,” “Every few years,” “Once a year,” and “Twice a year”) and those occurring more frequently than every 6 months (including “Monthly,” “Weekly,” and “Daily”). This grouping meaningfully categorized events as infrequent versus frequent, supporting relevant analytical comparisons.

These categorizations were implemented to optimize statistical testing and interpretability of ordinal Likert-scale data while preserving meaningful distinctions across response patterns.

### 3.4. Analytic Strategy

Descriptive statistics were calculated for participant demographics (age, sex, region, type of practice) and key questionnaire items. Continuous variables (age; Likert-scale scores for professional knowledge, resistance awareness, prevention knowledge, patient expectations, telemedicine prescribing frequency, and physical examination adherence) were summarized as mean ± standard deviation (SD) and range. Categorical variables (gender, practice type, region) were presented as counts and percentages.

Although Likert-scale data are inherently ordinal, we reported means and standard deviations alongside medians and interquartile ranges (IQR) for these variables in our descriptive statistics. This is a widely accepted convention in survey research to provide a more nuanced understanding of central tendency and data dispersion. However, to maintain statistical rigor, these variables were strictly treated as ordinal and non-normally distributed for all inferential analyses, utilizing appropriate non-parametric tests (Mann–Whitney U and Kruskal–Wallis).

Data were analyzed using non-parametric methods due to the non-normal distribution of variables, as assessed by the Shapiro–Wilk test (*p* < 0.001). Differences between two independent groups were evaluated using the Mann–Whitney U test [24]. Comparisons involving more than two independent groups were conducted with the Kruskal–Wallis test [25]. When the Kruskal–Wallis test indicated a significant difference, post hoc pairwise comparisons with Bonferroni correction were applied to adjust for multiple testing [26].

All analyses were conducted using IBM SPSS statistical software (version 27.0; Armonk, NY, USA) [27], with significance defined at *p* < 0.05.

## 4. Results

### 4.1. Demographic Characteristics

Of the 193 physicians initially recruited, 12 were excluded due to extensive incomplete responses or ineligibility, resulting in a final sample of 181 participants. Participants who omitted certain demographic questions or had occasional missing values within the main questionnaire were retained in the study, provided they completed the core sections of the survey. Naturally, any missing data points were excluded from the specific item-level analyses and are explicitly reported in the results. The mean age was 54.5 ± 12.8 years (range: 27–79). Female participants made up 51.9% (n = 94) of the sample, while males accounted for 48.1% (n = 87).

Regarding their place of work, 58.6% (n = 106) worked in cities (including county-level and regular towns), 27.6% (n = 50) in the capital, and 13.3% (n = 24) in villages or large villages, with 1 participant (0.5%) leaving this question blank (Table 1).

Regarding professional qualifications, 5.5% (n = 10) were residents in training without a specialist exam, 30.9% (n = 56) held one specialist qualification, 40.9% (n = 74) had two specialist qualifications, and 22.7% (n = 41) held three or more.

### 4.2. Practice Characteristics

Among the participants, 149 (82.3%) reported owning a primary care practice. Of those practice owners who specified their practice type (n = 109), 70.6% (n = 77) operated adult practices and 29.4% (n = 32) ran mixed (adult and pediatric) practices. None of our respondents had solely pediatric practice (a notable proportion of the total sample, 32.6% (n = 59), did not clarify the type of practice).

Regarding patient population size, 31.5% (n = 57) served fewer than 1500 patients, 30.4% (n = 55) served 1500–2000 patients, and 23.2% (n = 42) reported serving more than 2000 patients, while 14.9% (n = 27) did not provide an answer to this question (Table 2).

### 4.3. Professional Characteristics

Participants were assessed in six domains: knowledge regarding antibacterial therapy, knowledge about bacterial resistance, understanding of infectious diseases, attention to antibiotic prescribing indicators, perception of the importance of resistance, and perceived ability to act against resistance.

As shown in Table 3, the majority of respondents reported high self-assessed professional knowledge on antibiotics, with 106 participants (58.9%) scoring 4 points and 39 participants (21.7%) scoring 5 points. Regarding knowledge of bacterial resistance, 46.7% (n = 84) scored 4 points, while 36.7% (n = 66) had low scores (0–3 points), and 1.1% (n = 2) did not provide an answer. Understanding of infectious disease mechanisms was generally strong, with 38.9% (n = 70) achieving the highest score. In contrast, only 17.8% (n = 32) scored 5 points for regularly monitoring their antibiotic prescribing indicators, while 59.4% (n = 107) reported low attention (0–3 points) in this area. Almost all participants (81.7%, n = 147) considered antibiotic resistance to be a very important public health problem, but only 52.2% (n = 94) felt strongly capable of taking action against resistance.

### 4.4. Patient Expectations and Prescribing Behaviors

Regarding patient expectations, 145 physicians (80.6%) reported that their patients typically expect to receive an antibiotic prescription for their symptoms. Only 5 (2.8%) indicated that they do not take the time to educate patients about the appropriate use of antibiotics. In total, 115 (63.9%) stated that their patients express dissatisfaction when antibiotics are not prescribed for their symptoms. In total, 70 (38.9%) participants reported having encountered patients who initiated antibiotic therapy without prior medical consultation, while 89 (49.4%) noted that their patients visit out-of-hours services to obtain antibiotic prescriptions.

A majority of 157 doctors (87.2%) stated that they do not yield to patient pressure by prescribing antibiotics when they are not clinically indicated. In total, 57 (37.7%) participants believed their patients are aware of the effects and side effects of antibiotics, whereas 168 (93.3%) reported that their patients adhere to the prescribed therapy. In total, 53 (29.4%) indicated that their patients request specific brands of antibiotics, and 167 (92.8%) noted that patients are generally receptive to their professional advice. Furthermore, 154 general practitioners (85.6%) reported that they do not prescribe antibiotics following telephone consultations, and 173 (96.1%) stated that they perform a thorough physical examination before issuing an antibiotic prescription (Table 4).

### 4.5. Frequency of Antibiotic-Related Events

In total, 128 participants (71.1%) reported having experienced conflicts with patients over not prescribing antibiotics within the past six months. Among them, 8 physicians (4.4%) encountered such conflicts daily, while 33 reported weekly conflicts. Despite this, only 26 doctors (14.4%) indicated that patients had left their practice due to disagreements over antibiotic prescriptions in the past six months, whereas 80 (44.2%) stated that this had never occurred in their practice.

Furthermore, 65 general practitioners (36.1%) admitted to prescribing antibiotics “just in case” during the last six months, and 101 (56.1%) reported prescribing antibiotics to complete their patients’ self-medication during the same period (Table 4).

### 4.6. Associations Between Demographics and Professional Characteristics

No significant associations were found between self-reported knowledge and attitudes towards antibiotics (professional characteristics) and gender, number of patients, or place of residence. Furthermore, Kruskal–Wallis tests revealed that age and year of graduation showed no significant associations across any of the six assessed professional domains (Table 5).

When examining the number of specialist exams and self-reported knowledge regarding antibacterial therapy, a significant overall difference was found (*p* = 0.003). Descriptive statistics showed that GPs with low scores (0–3 points) and medium scores (4 points) both had a median of 2 [IQR: 1–2] specialty qualifications. GPs with high scores (5 points) had a higher upper quartile, with a median of 2 [IQR: 2–3] specialty qualifications. Post hoc pairwise comparisons with Bonferroni correction revealed no significant difference in the number of specialties between GPs with low and medium scores (adjusted *p* = 1.000). However, significant differences were observed between low and high scores (adjusted *p* = 0.007) and between medium and high scores (adjusted *p* = 0.007). This indicates that GPs with high self-assessed knowledge scores on antibacterial therapy held significantly more specialty qualifications than those with lower scores.

Similar associations were found between the number of specialty qualifications and the self-assessed knowledge of bacterial resistance (*p* = 0.016). Following post hoc tests with Bonferroni correction, a significant difference was observed specifically between low (0–3 points) and high (5 points) scores (adjusted *p* = 0.022), indicating that GPs with high self-assessed knowledge of bacterial resistance held more specialty qualifications than those with low scores. The difference between medium and high scores was not statistically significant (adjusted *p* = 0.647).

Regarding awareness of the prevention and pathomechanism of infectious diseases, the overall Kruskal–Wallis test indicated a marginally significant difference based on the number of specialist exams (*p* = 0.038). However, after applying the Bonferroni correction for multiple post hoc comparisons, none of the pairwise group differences reached statistical significance (adjusted *p* = 1.000, *p* = 0.074, and *p* = 0.126, respectively).

Finally, there were no significant differences in the number of specialty qualifications across groups for GPs’ monitoring of antibiotic prescribing indicators (*p* = 0.566), perception of the importance of antibiotic resistance (*p* = 0.541), or perceived ability to take action against resistance (*p* = 0.302).

### 4.7. Associations Between Demographics and Patient Expectations and Prescribing Behaviors

No significant associations were found between patient and practice characteristics and the gender of the doctor, number of patients, or place of residence.

Mann–Whitney U tests were conducted to examine differences in age and number of specialty qualifications between GPs grouped by their responses to the item “My patients expect antibiotics”.

No significant differences were observed in age (U = 2009.000, Z = −1.911, *p* = 0.056) or in the number of specialty qualifications (U = 2137.500, Z = −1.529, *p* = 0.126) between GPs who reported low versus high patient expectations.

After examining differences between GPs who reported that they rarely educate patients and those who regularly educate patients, we found no significant differences between the groups in age (U = 330.000, Z = −0.936, *p* = 0.349). However, a significant difference was observed in the number of specialty qualifications (U = 214.000, Z = −2.057, *p* = 0.040). GPs who reported rarely educating patients had a higher mean number of specialties (mean = 2.60, median = 3.00) compared to those who reported regularly educating patients (mean = 1.84, median = 2.00). This suggests that GPs with more specialty qualifications may be less involved in direct patient education regarding antibiotic use.

Our findings revealed no significant correlations between age or number of specialty qualifications and the other domains of this part of our questionnaire.

### 4.8. Associations Between Demographics and Frequency of Antibiotic-Related Events

Regarding conflicts with patients over not prescribing antibiotics, significant differences were observed in age (U = 2299.500, Z = −3.248, *p* = 0.001) and number of specialty qualifications (U = 2553.500, Z = −2.585, *p* = 0.010).

GPs who reported less frequent conflicts (less frequent than every 6 months) were younger (mean age = 52.57, median = 54.00) compared to those who experienced conflicts more frequently (more frequent than every six months) (mean age = 59.48, median = 59.50). The number of specialty qualifications was also slightly higher among GPs with more frequent conflicts (mean = 2.12, median = 2.00) than among those reporting less frequent conflicts (mean = 1.76, median = 2.00).

No significant differences were observed for the other patient-related prescribing behaviors, including just-in-case prescribing, patients leaving the practice due to conflicts over prescriptions, or prescribing antibiotics to complete a course initiated through self-medication (Table 6).

## 5. Discussion

Our cross-sectional study provides important insights into the complex relationship between general practitioners’ attitudes, patient expectations, and antibiotic prescribing practices in Hungarian primary care. This study identifies several possible areas of interest that warrant further investigation into how professional and patient-related factors intersect in primary care.

### 5.1. The Paradox of Awareness Versus Action

A striking finding of our study is the apparent disconnect between physicians’ awareness of antimicrobial resistance as a public health concern and their perceived ability to address it effectively. While almost all participants (81.7%, n = 147) considered antibiotic resistance to be a very important public health problem, only 52.2% (n = 94) felt strongly capable of taking action against resistance. The gap between awareness and self-efficacy may reflect systemic barriers, resource limitations, or inadequate training in antimicrobial stewardship strategies that extend beyond individual clinical decision-making [28].

### 5.2. Professional Experience and Clinical Confidence

Our analysis revealed a significant association between the number of specialty qualifications and self-assessed knowledge regarding antibiotic therapy. GPs with high self-assessed knowledge scores on antibiotic therapy held more specialty qualifications than those with low or medium scores. This finding suggests that additional professional training and specialization may contribute to physicians’ confidence in their antimicrobial prescribing decisions. However, this increased confidence may represent a double-edged phenomenon, as it could potentially lead to overconfidence in clinical judgment or reduced receptivity to external guidance and continuing education initiatives [29,30].

Internationally, this aligns with evidence of “optimism bias” or “unrealistic optimism” observed in primary care settings. For instance, research conducted in Sweden, a country with relatively low prescribing rates, indicates that physicians and nurses frequently rate their own guideline compliance and knowledge as significantly higher than that of their peers, even when objective assessments reveal gaps in actual knowledge regarding antibiotic efficacy [29].

### 5.3. The Resilience of Professional Standards Despite Conflict

Despite the high frequency of patient conflicts regarding antibiotic prescriptions, with 71.1% of physicians reporting such conflicts within the past six months, the vast majority (87.2%) stated that they do not yield to patient pressure by prescribing antibiotics when clinically contraindicated. This finding contradicts common assumptions about the inevitability of defensive prescribing in response to patient demands and suggests that Hungarian GPs maintain professional boundaries despite interpersonal challenges [31]. However, these self-reported figures may be subject to social desirability bias.

Furthermore, only 14.4% of physicians reported that patients had left their practice due to disagreements over antibiotic prescriptions, indicating that conflicts over prescribing decisions do not necessarily result in irreparable damage to the doctor-patient relationship or practice sustainability [32].

### 5.4. Age, Experience, and Conflict Management

The relationship between physician age, specialty qualifications, and conflict frequency presents an intriguing pattern. Our results show that GPs who reported less frequent conflicts were younger (mean age = 52.57) compared to those who experienced more frequent conflicts (mean age = 59.48), while physicians with more specialty qualifications reported more frequent conflicts. This counterintuitive finding challenges the assumption that greater experience and specialization necessarily lead to smoother patient interactions. Several explanations merit consideration: younger physicians may be more adaptable to current communication strategies, may have received more recent training in patient-centered care approaches, and may be less burnt out than their older colleagues [33]. Conversely, more experienced physicians with multiple specialties may attract more demanding patients or maintain stricter adherence to evidence-based guidelines regardless of patient preferences [34].

### 5.5. Specialty Training and Patient Education

Statistical analysis revealed that GPs with a higher number of specialty qualifications reported less frequent involvement in patient education regarding antibiotic use.

Physicians who rarely educate patients have a higher mean number of specialties (mean = 2.60) compared to those who regularly educate patients (mean = 1.84). This pattern may reflect several factors, including time constraints, assumptions that patients should already possess basic knowledge, or a focus on technical medical aspects rather than patient communication. This finding raises concerns about the potential for creating gaps in patient education, precisely among physicians who may possess the most comprehensive knowledge about antimicrobial therapy.

This behavior aligns with observations of US pediatric surgeons, who often view their early education and fellowship training as the definitive guides for their practice. Many such specialists report a lack of formal training in modern prescribing communication and exhibit resistance to changing clinical habits they have used successfully for decades. In this context, the reduced emphasis on education among highly specialized GPs may represent a reliance on the technical mastery established during their initial training, rather than an adoption of the communication-centered requirements of contemporary antibiotic stewardship [35].

It is important to note that while our analysis identified statistically significant differences in the associations between the recency of patient conflicts and the number of specialty qualifications held by the GP, the absolute differences between these groups were marginal (e.g., a mean of 2.12 versus 1.76 specialties, with identical medians). Therefore, these specific findings should be interpreted with caution regarding their practical or clinical significance. Statistical significance in these instances indicates a measurable pattern but may correspond to small effect sizes that do not necessarily translate to major clinical or systemic differences in real-world practice.

### 5.6. The Self-Medication Paradox

One of the most concerning findings relates to physicians’ reports of high patient adherence (93.3%) alongside frequent prescribing to complete patient-initiated antibiotic courses (56.1%). This paradox highlights a significant public health concern, as antibiotics are not available over the counter in Hungary, raising questions about how patients obtain these medications for self-treatment. According to a 2010 article, possible explanations include the use of leftover medications from previous prescriptions, sharing medications among family members, or using previously “just-in-case” prescribed antibiotics [36].

These contradictory findings also point toward a critical methodological limitation. The reliance on self-reported data introduces the risk of social desirability bias, where respondents may have felt compelled to report high adherence rates to appear compliant. Consequently, the high adherence figure (93.3%) should be interpreted with caution, as it may be artificially inflated by participants’ desire to provide socially acceptable answers.

The practice of prescribing antibiotics to complete self-initiated therapy is particularly problematic, as it often involves inappropriate antibiotic selection, inadequate dosing, or treatment of conditions that may not warrant antibiotic therapy, thereby contributing to antimicrobial resistance while potentially reinforcing inappropriate self-medication behaviors [37]. The frequent completion of patient-initiated antibiotic courses suggests that resistance to inappropriate prescribing may, in practice, be conditional rather than absolute. Once antibiotic therapy has already been initiated, often outside medical supervision, general practitioners may perceive completion of the course as the lesser of two harms, aiming to reduce the risk of underdosing and resistance selection. While clinically understandable, this practice effectively legitimizes unsupervised antibiotic use and may inadvertently reinforce future self-medication behaviors.

Although antibiotics are prescription-only in Hungary, the reported prevalence of self-medication (38.9%) and frequent completion of patient-initiated courses suggest that a substantial amount of leftover antibiotics may be circulating in the community. Likely sources include unused medication from previous prescriptions and intra-household sharing, phenomena well documented in other European settings. For instance, a 2015 study in Lithuania found that 27.8% of respondents had used antibiotics without a prescription [38]. This informal availability represents a structural vulnerability in antimicrobial stewardship that extends beyond the point of prescribing [39,40].

An additional inconsistency emerges between the high proportion of physicians who report patient adherence to prescribed therapy (93.3%) and the substantial prevalence of self-medication (38.9%). This misalignment may contribute to overly optimistic assumptions regarding patient behavior, potentially limiting the effectiveness of educational interventions when physicians underestimate the likelihood of unsupervised antibiotic use [38].

### 5.7. Limited Impact of Monitoring Systems

The finding that only 17.8% of physicians regularly monitor their antibiotic prescribing indicators, while 59.4% report low attention to this area, suggests that existing Hungarian performance-based incentive systems may have limited effectiveness in driving change in prescribing behavior. This observation raises important questions about the design and implementation of antimicrobial stewardship programs in primary care settings. The low engagement with monitoring systems may reflect insufficient feedback mechanisms and funding, inadequate integration with clinical workflows, or physicians’ perception that professional judgment should take precedence over administrative metrics [41].

In contrast to the Hungarian indicator system, the United Kingdom has seen more measurable success through a multifaceted approach. The volume of antibiotics prescribed in the UK fell from 20 Defined Daily Doses (DDDs) per 1000 inhabitants/day in 2013 to 18 DDDs in 2023. This success is attributed not just to indicators, but to public awareness campaigns and a national 20-year vision for AMR. However, as of 2023, UK levels remain approximately double those of the Netherlands, with 9.6 DDDs in 2023, which is widely considered the gold standard for stewardship in Europe [42].

### 5.8. Adherence to Clinical Standards

Despite reporting frequent conflicts and pressure from patients, physicians demonstrate strong adherence to fundamental clinical practices, with 96.1% performing thorough physical examinations before antibiotic prescription and 85.6% avoiding antibiotic prescriptions during telephone consultations. These practices reflect appropriate clinical standards, suggesting that concerns about telemedicine leading to increased inappropriate prescribing may be less pronounced in Hungarian primary care [43,44]. However, the admission by 36.1% of physicians to prescribing antibiotics “just in case” indicates that defensive prescribing practices persist despite adherence to examination protocols [45].

### 5.9. Physician Characteristics and Prescribing Expectations

Notably, our study found no significant associations between patient expectations for antibiotics and physician characteristics such as age, gender, or number of specialty qualifications. This finding suggests that patient pressure for antibiotic prescriptions may be a universal phenomenon across different physician demographics rather than being concentrated among specific physician groups.

To further investigate this issue, we are currently conducting a study among the Hungarian population to assess antibiotic-related knowledge, attitudes, and expectations.

## 6. Limitations

Our study’s limitations should be acknowledged when interpreting these findings. First, while the cross-sectional design of this study prevents the determination of causality, the robust associations identified between physician characteristics and prescribing behaviors provide a critical foundation for understanding how professional dynamics and patient pressures intersect.

Second, while the study focused on the northeastern region of Hungary, potentially limiting broader generalizability, the recruitment strategy served to mitigate several common biases. By conducting the study during statutory, mandatory medical education courses, we accessed a broad cross-section of the professional population, including those less likely to participate in voluntary surveys. This approach effectively mitigated the selection bias typically associated with convenience sampling in educational settings. However, the geographic focus means that while the sample is representative of the regional GP workforce, regional variations in healthcare infrastructure across Hungary might influence results elsewhere.

Third, the purpose-built nature of the survey instrument presents both a strength and a limitation. A new tool was necessitated by the unique socio-cultural and structural specificities of the Hungarian primary care system, which existing international instruments do not fully capture. Although the tool demonstrated acceptable internal consistency (α = 0.72), it has not yet undergone formal psychometric validation. As such, this study represents a critical exploratory step toward the future validation of a context-specific measurement tool.

Furthermore, regarding data analysis, the necessary categorization and dichotomization of ordinal Likert-scale responses, while performed to optimize statistical testing and facilitate clear interpretation, presents a limitation. This methodological choice inherently risks a loss of granular information and may obscure meaningful, subtle gradients in physician attitudes, knowledge, and prescribing behaviors. Future studies utilizing the validated version of this instrument should consider incorporating advanced psychometric modeling to explore these nuanced gradients more deeply.

Finally, as with all self-reported data, our findings may be influenced by social desirability bias, particularly regarding sensitive topics like “just-in-case” prescribing or yielding to patient pressure. The use of paper-based questionnaires also led to sporadic missing data, notably regarding the “Type of Practice” variable, likely due to visual layout issues. We addressed this by employing an item-by-item analysis to preserve statistical power, and all missing values are explicitly reported to maintain transparency.

## 7. Conclusions

This study characterizes the Hungarian primary care landscape as one defined by high awareness of antimicrobial resistance but persistent difficulty in translating this knowledge into effective action. While physicians demonstrate resilience in maintaining professional standards despite frequent patient conflicts, several concerning patterns emerge that warrant targeted interventions.

The paradoxical relationship between high reported patient adherence and the frequent completion of self-initiated antibiotic therapy represents a critical area for public health intervention. This phenomenon highlights a structural vulnerability in stewardship where leftover medications or shared supplies circulate within the community, necessitating coordinated interventions such as revised package sizes and national awareness campaigns to interrupt the cycle of unsupervised antibiotic use.

Limited engagement with antibiotic prescribing indicator monitoring suggests that current antimicrobial stewardship approaches may be insufficient. Performance-based monitoring alone appears inadequate, and stewardship strategies may require redesign to better integrate with routine clinical workflows. Integrating real-time, guideline-based feedback into electronic prescribing systems could support decision-making at the point of care while providing meaningful feedback without increasing administrative burden.

The observation that more experienced physicians report more frequent conflicts, despite greater clinical knowledge, suggests that communication skills training should be an ongoing component of professional development rather than a one-time educational intervention. Similarly, the finding that physicians with multiple specialty qualifications report less frequent patient education indicates that effective communication cannot be assumed to arise solely from clinical expertise. Structured communication training should therefore be considered a core and recurring element of continuing medical education.

Although adherence to core clinical standards remains high, the frequent experience of conflict underscores the need for clearer, nationally adapted antibiotic prescribing guidelines. Such guidelines could provide both clinical and legal support for general practitioners, reducing defensive “just-in-case” prescribing while strengthening stewardship efforts.

Ultimately, these results emphasize that addressing inappropriate antibiotic use requires a comprehensive approach targeting both individual prescriber behavior and the broader healthcare system. Although the resilience of professional standards provides an encouraging foundation, overcoming deep-seated patient expectations and self-medication behaviors will necessitate sustained, multi-level interventions involving patients, providers, and healthcare system administrators alike.

## Figures and Tables

**Table 1:** Demographic characteristics of the participants.

Variable	N	%
Gender		
Male	87	48.1
Female	94	51.9
Place of work		
Capital (Budapest)	50	27.6
City (county-level or other)	106	58.6
Village/Large village	24	13.3

Note: N = 181 for all categories. Missing data are reported as ‘No response’ where participants chose not to disclose demographic information but were retained due to completing the main survey.

**Table 2:** Characteristics of GP practices.

Variable	N	%
Own GP practice		
Yes	149	82.3
No	31	17.1
Type of practice		
Adult	77	42.5
Mixed	32	17.7
None	13	7.2
No response	59	32.6
Patient population size		
<1500	57	31.5
1500–2000	55	30.4
>2000	42	23.2
No response	27	14.9

Note: N = 181 for all categories. Missing responses are included to ensure full transparency of the retained sample.

**Table 3:** Distribution of scores across domains among GPs.

Domain	0–3 Points (N/%)	4 Points (N/%)	5 Points (N/%)	No Response (N/%)
Knowledge regarding antibacterial therapy	35 (19.4%)	106 (58.9%)	39 (21.7%)	0 (0.0%)
Knowledge of bacterial resistance	66 (36.7%)	84 (46.7%)	28 (15.6%)	2 (1.1%)
Understanding of infectious diseases	33 (18.3%)	77 (42.8%)	70 (38.9%)	0 (0.0%)
Monitoring indicators	107 (59.4%)	41 (22.8%)	32 (17.8%)	0 (0.0%)
Perception of resistance importance	10 (5.6%)	23 (12.8%)	147 (81.7%)	0 (0.0%)
Perceived ability to act	35 (19.4%)	51 (28.3%)	94 (52.2%)	0 (0.0%)

Note: Total N = 180 for this section of the questionnaire. Percentages may not sum to exactly 100.0% due to standard rounding.

**Table 4:** Patient expectations and prescribing behaviors.

Domain	No	Yes	No Response (N/%)
My patients expect antibiotics	35 (19.4%)	145 (80.6%)	1 (0.6%)
I educate patients	5 (2.8%)	175 (97.2%)	1 (0.6%)
Patients dissatisfied over no AB prescription	65 (36.1%)	115 (63.9%)	1 (0.6%)
Self-medication	110 (61.1%)	70 (38.9%)	1 (0.6%)
Out-of-hours services for AB	91 (50.6%)	89 (49.4%)	1 (0.6%)
I yield to patient pressure	157 (87.2%)	23 (12.8%)	1 (0.6%)
My patients know effects and side effects	123 (68.3%)	57 (31.7%)	1 (0.6%)
My patients follow therapy I prescribe	12 (6.7%)	168 (93.3%)	1 (0.6%)
My patients request a specific brand/active ingredient	127 (70.6%)	53 (29.4%)	1 (0.6%)
My patients are receptive of my advice	13 (7.2%)	167 (92.8%)	1 (0.6%)
Telemedicinal prescription	154 (85.6%)	26 (14.4%)	1 (0.6%)
Physical examination before prescription	7 (3.9%)	173 (96.1%)	1 (0.6%)
	**>6 months (N/%)**	**<6 months (N/%)**	**No response (N/%)**
Conflict over not prescribing	52 (28.9%)	128 (71.1%)	1 (0.6%)
Just in case prescription	115 (63.9%)	65 (36.1%)	1 (0.6%)
Patients leave praxis due to conflict over prescription	154 (85.6%)	26 (14.4%)	1 (0.6%)
Prescribing to complete self-medication	79 (43.9%)	101 (56.1%)	1 (0.6%)

**Table 5:** Associations between demographics and professional characteristics.

Independent Variable (Self-Reported Statements)	Dependent Variable	Kruskal-Wallis *p*-Value	Median [IQR]	Bonferroni Correction (Adj. Sig.)
1. My professional knowledge is adequate regarding antibacterial therapies. 1- 0–3 points 2- 4 points 3- 5 points	Age (years)	0.653	1: 58 [48–65] 2: 57 [41–65] 3: 57 [47–64]	-
	Year of graduation	0.608	1:1991 [1984–2001] 2:1993 [1985–2008] 3: 1992 [1986–2001]	-
	Number of specialist exams	**0.003**	1: 2 [1–2] 2: 2 [1–2] 3: 2 [2–3]	1 vs. 2: *p* = 1.000 1 vs. 3: ***p* = 0.007** 2 vs. 3: ***p* = 0.007**
2. My knowledge regarding bacterial resistance is adequate. 1- 0–3 points 2- 4 points 3- 5 points	Age (years)	0.465	1: 53 [42–65] 2: 58 [44–64] 2: 62 [47–66]	-
	Year of graduation	0.470	1: 1996 [1986–2007] 2: 1993 [1987–2006] 3: 1989 [1982–2003]	-
	Number of specialist exams	**0.016**	1: 2 [1–2] 2: 2 [1–2.5] 3: 2 [2–3]	1 vs. 2: *p* = 0.127 1 vs. 3: ***p* = 0.022** 2 vs. 3: *p* = 0.647
3. I am aware of the prevention and pathomechanism of infectious diseases. 1- 0–3 points 2- 4 points 3- 5 points	Age (years)	0.294	1: 58 [45–68] 2: 55 [41–63] 3: 58 [47–65]	-
	Year of graduation	0.217	1: 1991 [1982–2005] 2: 1994 [1987–2008] 3: 1991 [1983–2005]	-
	Number of specialist exams	**0.038**	1: 2 [1–2] 2: 2 [1–2] 3: 2 [2–3]	1 vs. 2: *p* = 1.000 1 vs. 3: *p* = 0.074 2 vs. 3: *p* = 0.126
4. I pay attention to my antibiotic indicator score. 1- 0–3 points 2- 4 points 3- 5 points	Age (years)	0.145	1: 55 [41–64] 2: 59 [52–64] 3: 59 [44–66]	-
	Year of graduation	0.212	1: ~1995 [1985–2008] 2: ~1991 [1986–1997] 3: ~1989 [1983–2006]	-
	Number of specialist exams	0.566	1: 2 [1–3] 2: 2 [1–2] 3: 5 [1–2]	-
5. I consider antibiotic resistance an important public health problem. 1- 0–3 points 2- 4 points 3- 5 points	Age (years)	0.690	1: 54 [45–73] 2: 55 [44–63] 3: 58 [44–64]	-
	Year of graduation	0.739	1: 1994 [1979–2005] 2: 1993 [1988–2004] 3: 1992 [1985–2006]	-
	Number of specialist exams	0.541	1: 2 [1–2] 2: 1 [1–2] 3: 2 [1–2]	-
6. I can take action against the development of antibiotic resistance. 1- 0–3 points 2- 4 points 3- 5 points	Age (years)	0.877	1: 53 [44–64] 2: 58 [44–66] 3: 57 [44–64]	-
	Year of graduation	0.889	1: ~1995 [1986–2005] 2: ~1992 [1983–2006] 3: ~1992 [1985–2004]	-
	Number of specialist exams	0.302	1: 2 [1–2] 2: 2 [1–2] 3: 2 [1–2]	-

**Table 6:** Associations between demographics and frequency of antibiotic-related events.

Prescribing Event/Behavior	Variable	Less Frequent than 6 Months Mean ± SD Median [IQR]	More Frequent than 6 Months Mean ± SD Median [IQR]	*p*-Value
Conflict over not prescribing	Age (years)	52.57 ± 12.73 54.00 [21] (n = 128)	59.48 ± 11.81 59.50 [15] (n = 52)	**0.001**
	Number of specialties	1.76 ± 0.94 2.00 [1]	2.12 ± 0.98 2.00 [1]	**0.010**
“Just in case” prescription	Age (years)	54.74 ± 12.99 58.00 [21] (n = 115)	54.26 ± 12.64 54.00 [21] (n = 65)	0.759
	Number of specialties	1.90 ± 0.93 2.00 [2]	1.80 ± 1.02 2.00 [1]	0.329
Patients leave praxis due to conflict	Age (years)	55.17 ± 13.46 58.00 [21] (n = 104)	53.75 ± 11.97 55.00 [19] (n = 77)	0.390
	Number of specialties	1.87 ± 1.00 2.00 [2]	1.84 ± 0.92 2.00 [1]	0.811
Prescribing to complete self-medication	Age (years)	54.70 ± 13.02 58.00 [21] (n = 79)	54.47 ± 12.74 57.00 [21] (n = 101)	0.896
	Number of specialties	1.92 ± 0.98 2.00 [1]	1.81 ± 0.95 2.00 [1]	0.465

## Data Availability

The data presented in this study are available on request from the corresponding author. The data are not publicly available due to privacy restrictions.

## Abbreviations

The following abbreviations are used in this manuscript:

AMR	Antimicrobial resistance
GP	General Practitioner
NEAK	National Health Insurance Fund of Hungary
